# Evidence for Nanoparticle-Induced Lysosomal Dysfunction in Lung Adenocarcinoma (A549) Cells

**DOI:** 10.3390/ijms20215253

**Published:** 2019-10-23

**Authors:** Arnold Sipos, Kwang-Jin Kim, Constantinos Sioutas, Edward D. Crandall

**Affiliations:** 1 Will Rogers Institute Pulmonary Research Center and Hastings Center for Pulmonary Research, Keck School of Medicine, University of Southern California, Los Angeles, CA 90033-0906, USA; kjkim@usc.edu (K.-J.K.); edward.crandall@med.usc.edu (E.D.C.); 2 Division of Pulmonary, Critical Care and Sleep Medicine, Department of Medicine, Keck School of Medicine, University of Southern California, Los Angeles, CA 90033-0906, USA; 3 Department of Physiology and Neuroscience, Keck School of Medicine, University of Southern California, Los Angeles, CA 90089-9037, USA; 4 Department of Pharmacology and Pharmaceutical Sciences, School of Pharmacy, University of Southern California, Los Angeles, CA 90089-9121, USA; 5 Department of Biomedical Engineering, Viterbi School of Engineering, University of Southern California, Los Angeles, CA 90089-1111, USA; 6 Sonny Astani Department of Civil and Environmental Engineering, Viterbi School of Engineering, University of Southern California, Los Angeles, CA 90089-2531, USA; sioutas@usc.edu; 7 Department of Pathology, Keck School of Medicine, University of Southern California, Los Angeles, CA 90033-9092, USA; 8 Mork Family Department of Chemical Engineering and Materials Science, Viterbi School of Engineering, University of Southern California, Los Angeles, CA 90089-1211, USA

**Keywords:** autophagy, lysosome, lysosomal membrane permeability, mitochondria, pneumocyte

## Abstract

Background: Polystyrene nanoparticles (PNP) are taken up by primary rat alveolar epithelial cell monolayers (RAECM) in a time-, dose-, and size-dependent manner without involving endocytosis. Internalized PNP in RAECM activate autophagy, are delivered to lysosomes, and undergo [Ca^2+^]-dependent exocytosis. In this study, we explored nanoparticle (NP) interactions with A549 cells. Methods: After exposure to PNP or ambient pollution particles (PM0.2), live single A549 cells were studied using confocal laser scanning microscopy. PNP uptake and egress were investigated and activation of autophagy was confirmed by immunolabeling with LC3-II and LC3-GFP transduction/colocalization with PNP. Mitochondrial membrane potential, mitophagy, and lysosomal membrane permeability (LMP) were assessed in the presence/absence of apical nanoparticle (NP) exposure. Results: PNP uptake into A549 cells decreased in the presence of cytochalasin D, an inhibitor of macropinocytosis. PNP egress was not affected by increased cytosolic [Ca^2+^]. Autophagy activation was indicated by increased LC3 expression and LC3-GFP colocalization with PNP. Increased LMP was observed following PNP or PM0.2 exposure. Mitochondrial membrane potential was unchanged and mitophagy was not detected after NP exposure. Conclusions: Interactions between NP and A549 cells involve complex cellular processes leading to lysosomal dysfunction, which may provide opportunities for improved nanoparticle-based therapeutic approaches to lung cancer management.

## 1. Introduction

Nanoparticle (NP) exposure has been reported to induce stress in various cells and tissues [1,2,3,4]. The cellular response to NP exposure is dependent on the chemical (e.g., components and surface charge) and physical (e.g., shape and size) characteristics of NP [5,6,7,8,9]. Due to the heterogeneity of the physicochemical properties of NP, it is difficult to generalize their effects in cells. However, it has been reported that NP exposure of cells and tissues affects functions of various intracellular organelles (e.g., mitochondria and endoplasmic reticulum (ER)) [4,10,11,12]. The involvement of lysosomes in cellular stress (especially in lung alveolar epithelial cells (AEC)) in response to inhaled NP may be especially important in the pathogenesis of chronic lung disease.

Following internalization of foreign materials (e.g., proteins, bacteria, virus and (nano)particles), autophagy is activated as part of cellular defense mechanism(s) [13]. Autophagy is a catabolic process that helps maintain cellular homeostasis by removing excess, harmful, damaged, or foreign cellular components [14,15], although autophagy may play different roles in cancer versus normal cells, leading to either cell survival or death [16,17,18]. During autophagic processing, autophagosomes are formed to separate the target cytosolic component(s) from the remainder of the cell [19]. The autophagosome then delivers its cargo to the lysosome via autophagosome-lysosome fusion. Intracellular NP have been reported to activate autophagy [20], and NP localized inside (auto)lysosomes were shown to induce alteration(s) in lysosomal function [21]. Inhalation of airborne pollution particles (including those overlapping in size with nanoparticles) may contribute to chronic diseases (e.g., chronic obstructive pulmonary disease and pulmonary fibrosis) [22], although the mechanisms by which this occurs are not well understood [23,24].

A549 is a continuous cell line derived from a human pulmonary adenocarcinoma that is widely used as a model of mammalian lung alveolar epithelial type II cells. Various ultrastructural characteristics of A549 cells are similar to those in type II pneumocytes. Phospholipid composition of the A549 cell line has been shown to be similar to that of primary isolates of type II cells [25].

In this study, ambient air pollution particles (PM0.2, diameter <0.2 μm) and polystyrene nanoparticles (PNP) were used to investigate their intracellular handling/fate and their effects on A549 cells. PM0.2 contain nanoparticles (defined as particles whose size at least in one dimension is ≦100 nm) that may contribute to cellular health effects (including development of chronic lung diseases). PNP are engineered nanoparticles that are relatively nontoxic and non-metabolizable, making them suitable for studies of nanoparticle interactions/kinetics. They are useful especially for live cell imaging by taking advantage of their fluorescent labels. Utilizing NP (i.e., PM0.2 and 20 nm carboxylated PNP) and A549 as a model type II pneumocyte, we investigated in this study the internalization mechanisms, egress characteristics, and intracellular NP fate/handling/effects. We found that apical NP exposure of A549 cells leads to activation of autophagy and increased lysosomal membrane permeability (LMP) without mitochondrial dysfunction.

## 2. Results

### 2.1. Live Cell Imaging of Intracellular PNP in A549 Cells

**Figure 1:**
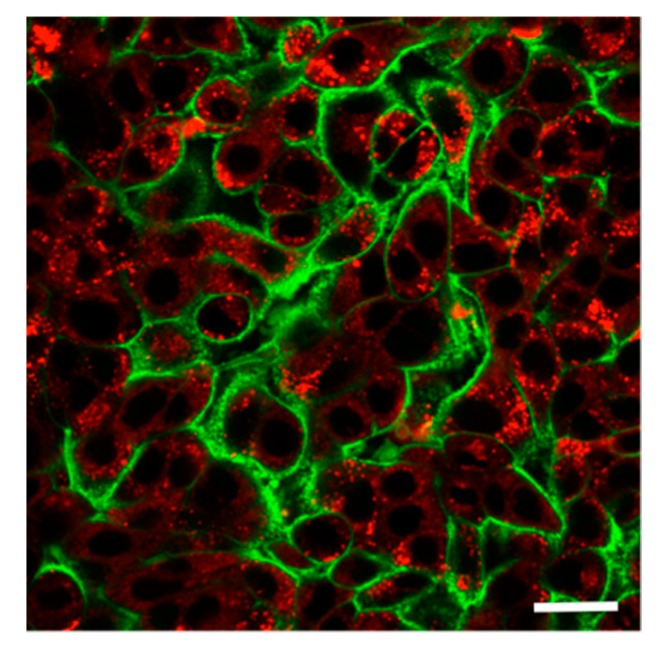
Intracellular accumulation of polystyrene nanoparticles (PNP) in A549 cells. Apical exposure of A549 cells to PNP at 80 μg/mL for 24 h led to accumulation of PNP (red) in intracellular vesicles, while diffuse distribution of PNP in cytosol was also seen. Plasma membranes of A549 cells were labeled by Dylight 488-conjugated tomato lectin (green). Scale bar is 10 μm.

At 24 h of apical PNP exposure of A549 cells, PNP (in red) accumulated in intracellular vesicles (Figure 1), while diffuse cytosolic PNP distribution was also seen. Plasma membranes of A549 cells were labeled by Dylight 488-conjugated tomato lectin (in green).

### 2.2. Mechanism(s) of PNP Entry into A549 Cells

**Figure 2:**
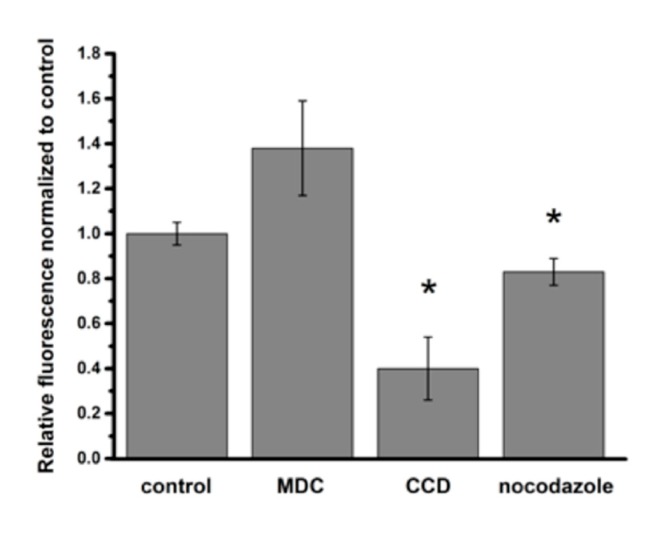
Relative changes in intracellular PNP content in A549 cells in the presence or absence (control) of endocytosis inhibitors after 24 h of apical PNP exposure. Monodansylcadaverine (MDC, an inhibitor of clathrin-mediated endocytosis) failed to decrease intracellular PNP content, whereas cytochalasin D (CCD, an inhibitor of macropinocytosis) and nocodazole (an inhibitor of microtubule polymerization) decreased intracellular PNP content by 60% and 17%, respectively. * *p* < 0.05 compared to control.

**Figure 3:**
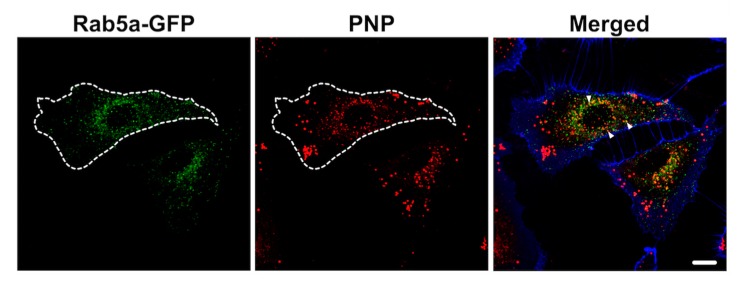
Colocalization of early endosome marker Rab5a-GFP with PNP in A549 cells. A549 cells were transduced for 2 h with an early endosome marker (Rab5a-GFP, green) and apically exposed thereafter to PNP (red) for 24 h. Colocalization (arrowheads, yellow) of PNP with Rab5a-GFP-positive vesicles was observed in some of the vesicles. Contours of cells were added (dotted lines) on the basis of the cell plasma membrane marker Dylight 405-conjugated tomato lectin (blue). Images are representative of 4–5 observations. Scale bar is 10 μm.

Nocodazole (an inhibitor of microtubule polymerization) decreased intracellular PNP content by 17% and cytochalasin D (CCD) decreased intracellular PNP content by 57%, indicating that macropinocytosis played a role in PNP entry into A549 cells (Figure 2). Clathrin-mediated endocytosis did not appear to be involved in PNP internalization, as monodansylcadaverine (MDC) failed to decrease intracellular PNP content (Figure 2). Further evidence for endocytic internalization of PNP into A549 cells was provided by colocalization of PNP with early endosomes that were pre-transduced with a Rab5-GFP vector (Figure 3).

### 2.3. PNP Egress from A549 Cells

**Figure 4:**
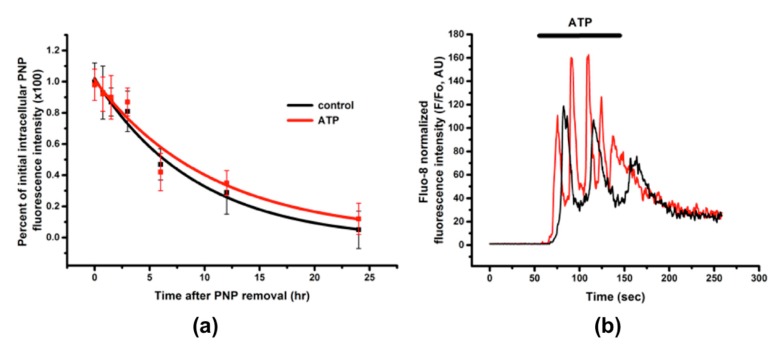
PNP egress from A549 cells. (**a**) A549 cells were apically exposed to PNP for 12 h, followed by washing with fresh culture fluid and assessing intracellular PNP content at designated time points for up to 24 h thereafter. When 10 μM ATP was applied apically to A549 cells at time zero and remained present throughout the entire experiment, no difference in PNP egress kinetics between control (no stimulation) and ATP-treated A549 cells during egress was observed. *n* = 4–6 for each time point. (**b**) Representative recording of oscillations in intracellular [Ca^2+^] detected upon 2.5 min presence of 10 μM ATP in the apical bathing fluid of A549 cells. Different colors represent intracellular [Ca^2+^] observed in two different A549 cells.

A549 cells were apically exposed to PNP (80 μg/mL) for 12 h, followed by washing with fresh cell culture fluid. Intracellular PNP content was assessed over time for up to 24 h thereafter. Intracellular PNP content of A549 cells decreased ~90% over 24 h (Figure 4). The egress profile in the continued presence of 10 μM apical ATP was not significantly different from that without ATP (Figure 4a), despite repeated elevations in cytosolic [Ca^2+^] due to brief (2.5 min) ATP stimulation (Figure 4b).

### 2.4. Intracellular NP Processing in A549 Cells

**Figure 5:**
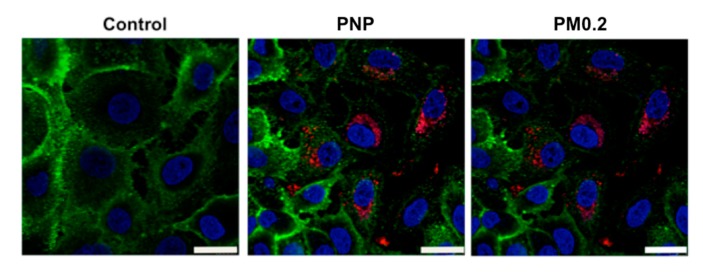
Apical nanoparticle (NP) exposure induced activation of autophagy in A549 cells. A549 cells were preincubated with chloroquine (40 μM, 30 min) and exposed thereafter to NP (PNP or ambient air pollution particles (PM0.2)) for 24 h in the continued presence of chloroquine, followed by assessment of LC3 expression by immunolabeling. LC3 expression (red) was detected in NP-exposed A549 cells. No or very low level of LC3 expression was found in control cells not exposed to NP. Plasma membranes of A549 cells were labeled by Dylight 488-conjugated tomato lectin (green), whereas nuclei were labeled by Hoechst 33342 (blue). Images are representative of 4–5 observations. Scale bars are 25 μm.

**Figure 6:**
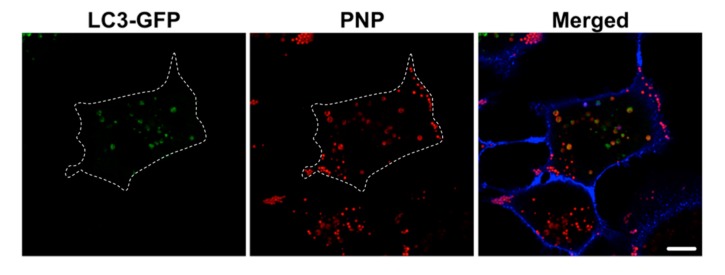
Colocalization of PNP with LC3-GFP in A549 cells. Following transduction of A549 cells with the autophagosome marker LC3-GFP construct for 2 h, cells were preincubated with chloroquine (40 μM) and apically exposed thereafter to PNP for 24 h in the continued presence of chloroquine. Colocalization of PNP (red) with LC3-GFP-positive vesicles (green) was observed. Contour of cell was added (dotted line) on the basis of the cell plasma membrane marker Dylight 405-conjugated tomato lectin (blue). Images are representative of 4–5 observations. Scale bar is 10 μm.

We investigated the involvement of autophagy in intracellular processing of NP. A549 cells were preincubated with an inhibitor (e.g., 40 μM chloroquine) of fusion of autophagosomes with lysosomes for 30 min prior to apical NP (PNP at 80 μg/mL or PM0.2 at 1 μg/mL) exposure, followed by exposure to NP (PNP or PM0.2) for 24 h in the continued presence of chloroquine. Immunolabeling for LC3-I/II of NP-exposed and chloroquine-treated A549 cells showed that the intracellular presence of NP led to activation of autophagy (Figure 5). This finding was confirmed in live LC3-GFP-transduced A549 cells (subsequently treated with chloroquine as well), where colocalization of PNP with LC3-GFP-positive intracellular vesicles (i.e., autophagosomes) was found (Figure 6).

**Figure 7:**
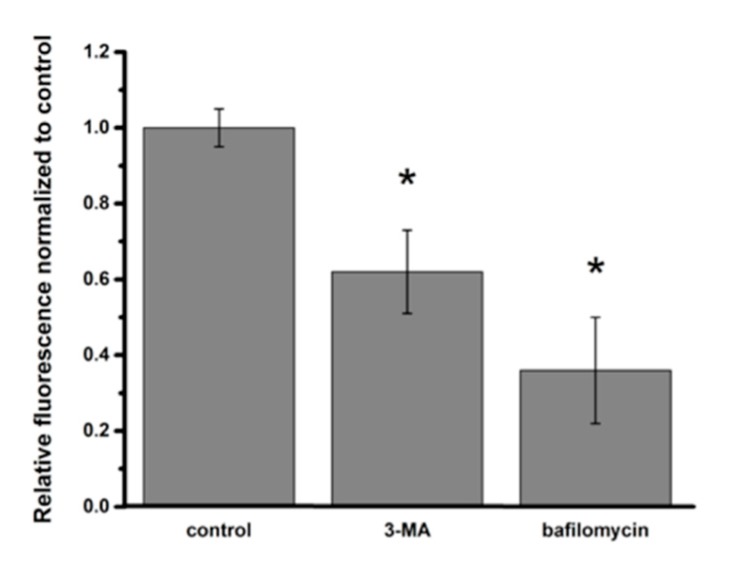
Effects of autophagy inhibitors on intracellular PNP content in A549 cells at 24 h post-exposure to PNP. Intracellular PNP content in A549 cells was reduced by 38% and 64%, respectively, when autophagosome formation was inhibited with 3-methyladenine (3-MA) or autophagosome-lysosome fusion was inhibited with bafilomycin. Data are normalized to control. * *p* < 0.05 compared to control.

Since autophagic processing of intracellular NP might affect the intracellular content of NP, we assessed intracellular PNP content in A549 cells in the presence of pharmacological inhibitors of autophagy. When autophagosome formation was inhibited by 3-methyladenine (3-MA), intracellular PNP content decreased by 38%, whereas impaired autophagic flux (in the presence of bafilomycin) resulted in a 64% decrease in intracellular PNP content, compared to that in control A549 cells at 24 h post-exposure (Figure 7).

### 2.5. Assessment of NP Exposure-Induced Lysosomal Dysfunction

**Figure 8:**
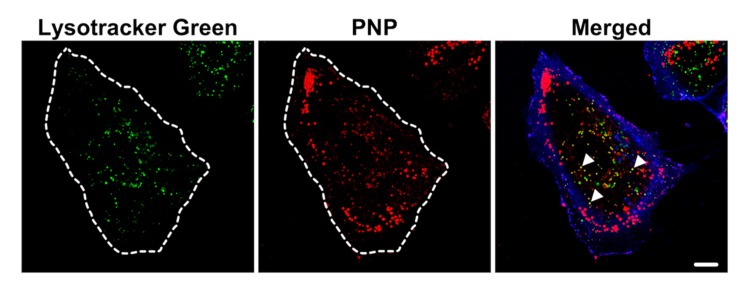
Colocalization of PNP with lysosomes in A549 cells. At 24 h post exposure to PNP, lysosomes in A549 cells were labeled with Lysotracker Green (green). Colocalization (yellow; arrowheads) can be seen between PNP (red) and Lysotracker Green in some of the vesicles in the perinuclear area. Plasma membrane of A549 cells was labeled by Dylight 405-conjugated tomato lectin (blue or shown with dotted line). Images are representative of 4–5 observations. Scale bar is 10 μm.

**Figure 9:**
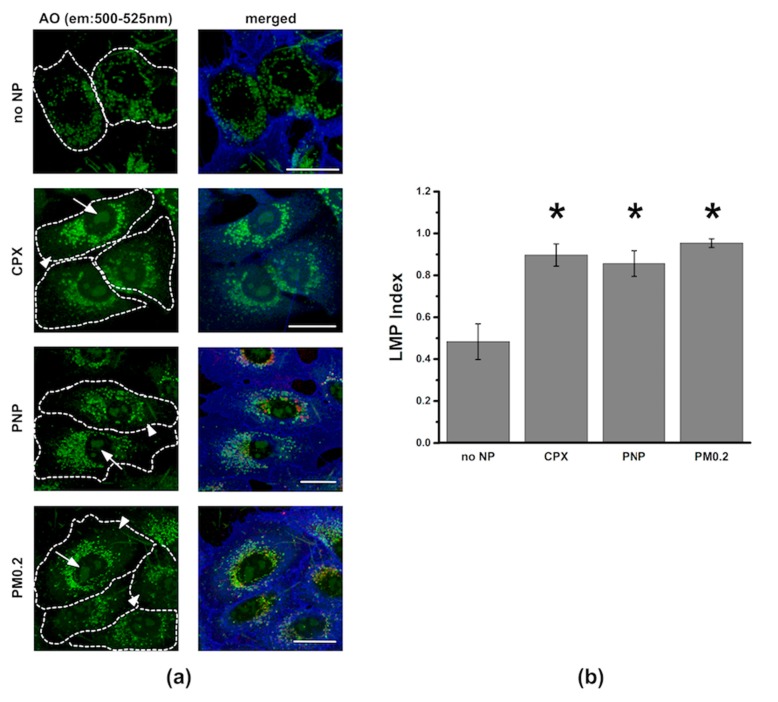
Detection of increased lysosomal membrane permeability (LMP) using acridine orange (AO) in A549 cells. (**a**) Increased LMP was detected when AO (green; ex/em: 488/500–525 nm) intensity in lysosomes was decreased along with increased AO intensity in cytoplasm and nucleus. In “no NP” panels, A549 cells showed virtually no detectable nuclear AO signal. A549 cells pretreated with ciprofloxacin (CPX, 150 μM for 24 h, without NP exposure) as positive control exhibited AO accumulation in both nucleus (arrow) and cytoplasm (arrowhead). NP (PNP or PM0.2) exposure also led to AO accumulation in cytoplasm and nucleus. Contours of cells were added (dotted line) on the basis of the cell plasma membrane marker Dylight 405-conjugated tomato lectin (blue). Scale bars are 20 μm. (**b**) When A549 cells were apically exposed to either PNP or PM0.2 for 24 h, increased LMP index (AO green fluorescence intensity in cytoplasm and nucleus/total cellular AO green fluorescence intensity) was found to be similar to that of positive control (CPX). *n* = 4–6. * *p* < 0.05 compared to control.

Colocalization of PNP (red) and lysosomes (green) was observed (Figure 8). PNP were also seen in A549 cells without colocalization with Lysotracker Green. PNP is non-metabolizable and likely to accumulate in lysosomes if intact lysosomal membranes are maintained. Acridine orange (AO) is known to accumulate in lysosomes and can be released upon lysosomal injury (e.g., derangement of lysosomal membrane integrity). When A549 cells were apically exposed to PNP or PM0.2 for 24 h, increased presence of AO in cytosol and nucleus was seen, indicating that LMP was increased (Figure 9).

### 2.6. NP Exposure and Mitochondrial Function in A549 Cells

**Figure 10:**
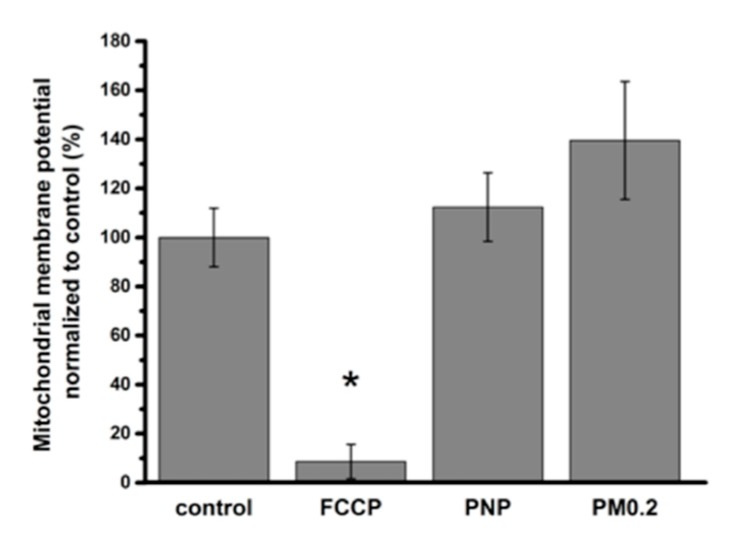
Effect of apical NP exposure for 24 h on mitochondrial membrane potential in A549 cells. Mitochondrial membrane potentials were normalized to the corresponding controls (i.e., not exposed to either NP or carbonyl cyanide 4-(trifluoromethoxy)phenylhydrazone (FCCP)). Mitochondrial membrane potential was nearly abolished in the presence of FCCP (1 μM, positive control), whereas it did not decrease following 24 h of apical exposure to PNP or PM0.2. * *p* < 0.05 compared to control.

**Figure 11:**
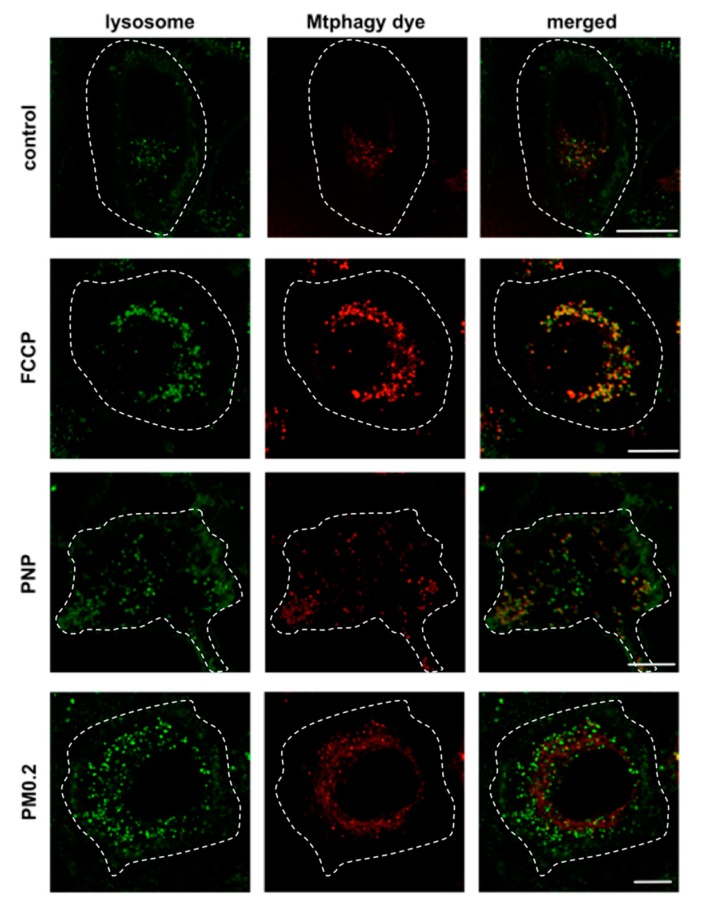
Absence of mitophagy in NP-exposed A549 cells. Mitophagy is detected on the basis of an increase in the fluorescent signal of the Mtphagy dye (in comparison to that of control; red) and colocalization of the Mtphagy dye with lysosomal indicator (LI; green) dye. In control (no exposure to PNP or PM0.2), the Mtphagy dye had weak fluorescence and minimal colocalization with lysosomes. After FCCP exposure (positive control), the Mtphagy dye exhibited strong fluorescence with extensive colocalization with the LI lysosomal marker dye. Exposure of A549 cells to either PNP or PM0.2 yielded weak fluorescence with the Mtphagy dye and showed minimal lysosomal colocalization of the Mtphagy dye, consistent with a low level (or relative absence) of mitophagy. Contours of cells were added (dotted line) on the basis of the cell plasma membrane marker Dylight 405-conjugated tomato lectin. Scale bars (25 μm) are shown in right panels only.

Mitochondria were labeled with the mitochondrial membrane potential sensitive fluorescent dye tetramethylrhodamine methyl ester (TMRM). When A549 cells were apically exposed to PNP or PM0.2 for 24 h, no change in mitochondrial membrane potential was observed (Figure 10). To confirm these data, we also used the Mtphagy dye, which is capable of staining damaged mitochondria (undergoing mitophagy and being delivered to lysosomes). No colocalization of the Mtphagy dye and LI lysosome marker dye was found, indicating that 24 h of apical exposure of A549 cells to PNP or PM0.2 did not lead to cellular stress involving mitochondrial dysfunction (Figure 11).

## 3. Discussion

Using a live cell imaging approach, we demonstrated that PNP are taken up into A549 cells, at least in part by macropinocytosis. Most (>90%) intracellular PNP content is released from A549 cells over 24 h, whereas mobilization of intracellular Ca^2+^ by ATP did not speed up PNP egress from A549 cells. Internalized NP (PNP or PM0.2) activated autophagy, delivering NP to lysosomes. The presence of NP in lysosomes led to increased lysosomal membrane permeability. Mitochondrial membrane potential was not affected and mitophagy was not observed in NP-exposed A549 cells.

### 3.1. Uptake of NP into A549 Cells

We previously reported that primary rat alveolar epithelial cell monolayers (RAECM) do not appear to utilize endocytic process(es) for PNP uptake, which are internalized mainly by diffusion-like entry into RAECM [26]. A549 cells, a lung adenocarcinoma cell line, differ from primary AEC in many ways, including the inability to form a barrier with high transepithelial resistance and utilization of macropinocytosis for NP uptake. PNP internalization that is not inhibited by cytochalasin D may take place via non-endocytic process(es) (e.g., diffusion across and/or poration by PNP of apical cell plasma membranes [27,28,29]). In addition, clathrin-mediated endocytosis is not involved in PNP uptake into A549 cells (Figure 2). The decrease in PNP uptake in the presence of nocodazole suggests that suppression of vesicle movement on an intracellular microtubular network is insufficient to entirely inhibit endocytosis. When A549 cells were transduced with the early endosome marker Rab5a-GFP, colocalization between early endosomes and PNP was found at 24 h post exposure to PNP, consistent with the presence of PNP endocytosis into A549 cells.

### 3.2. Egress of NP from A549 Cells

Intracellular PNP content decreased 90% over 24 h (Figure 4a) in egress experiments. Intracellular PNP was compartmentalized in A549 cells, as shown in Figure 1, with some PNP diffusely distributed in the cytoplasm. It has been reported that epithelial cells are capable of releasing the content of intracellular vesicles (e.g., lysosomes) [30,31]. For example, release of PNP localized in lysosomes of primary RAECM was dependent on elevations in cytosolic [Ca^2+^] [26]. By contrast, A549 cells stimulated with ATP led to oscillatory elevations in cytosolic [Ca^2+^] (Figure 4b) without altering the kinetics of PNP egress over 24 h (Figure 4a). In A549 cells, it is possible that lysosomal PNP leaked into cytosol and may not be available to exit primarily via lysosomal exocytosis.

### 3.3. Activation of Autophagy in Intracellular Processing of NP in A549 Cells

Autophagy is a defense mechanism aiming to maintain cellular homeostasis. The presence of NP in AEC has been reported to activate autophagy in primary RAECM [26]. In order to identify the involvement of autophagy in intracellular fate of internalized NP in A549 cells, we sought to detect the expression of microtubule-associated protein 1A/1B light chain 3B (LC3) protein. LC3 is localized to the phagophore membrane, followed by localization in inner and outer membranes of autophagosomes. Upon fusion of autophagosomes with lysosomes (i.e., autophagic flux), LC3 localized at the inner membrane of (auto)lysosomes is degraded by lysosomal proteolysis. LC3-GFP fusion protein expression is used widely to identify autophagosomes, with the caveat that GFP loses its fluorescence in the lysosomal acidic environment [32,33]. Because the autophagy process is relatively fast [34], there is only a small detection window (before autophagosomes fuse with lysosomes) for observing LC3-GFP, necessitating inhibition of autophagic flux (e.g., with chloroquine or bafilomycin to block the fusion of autophagosomes with lysosomes). In the presence of chloroquine, we observed colocalization of PNP with LC3-GFP after 24 h of apical PNP exposure, indicating that autophagy plays a role in cellular handling of NP in A549 cells (Figure 6). Further evidence for autophagy activation in NP-exposed A549 cells was obtained by immunolabeling of LC3-positive vesicles in NP-exposed A549 cells (Figure 5). PM0.2-exposed primary RAECM exhibited autophagy activation (i.e., expression of LC3) as well.

We observed that interference with autophagy led to decreased intracellular PNP content in A549 cells (Figure 7). When autophagic flux was blocked, autophagosomal content could not be delivered to lysosomes for further processing, as discussed below. Therefore, it is conceivable that the observed lower intracellular PNP content might be related to the decrease in lysosomal PNP localization. It cannot be ruled out that yet-unknown feedback mechanism(s) between autophagy and endocytic process(es) might also play a role. When we performed PNP uptake experiments in primary RAECM in which autophagy was inhibited with 3-MA or bafilomycin, intracellular PNP content was decreased by ~80 and ~50%, respectively [26]. In contrast to primary RAECM, the inhibition in A549 cells of autophagic flux resulted in greater loss in intracellular PNP content (64%) compared to inhibition of autophagosome formation (38%). Although we do not have a clear explanation for this difference between primary RAECM and A549 cells, it may indicate that A549 cell autophagy is regulated differently (especially by 3-MA [35]) from autophagy in primary AEC [36,37]. Lysotracker Green-negative intracellular vesicles that contain PNP may include amphisomes [20,38]. It is possible that the lysosomal dysfunction found in NP-exposed A549 cells (discussed below) also contributed to the difference in intracellular PNP content found in the presence of pharmacological inhibitors of autophagy.

### 3.4. Lysosomal Dysfunction in NP-Exposed A549 Cells

We found relatively low PNP content in lysosomes of A549 cells at 24 h post exposure (Figure 8). Most PNP resided in Lysotracker Green-negative vesicles, indicating that the PNP-filled vesicles were primarily non-lysosomal vesicles. This finding might explain why ATP stimulation failed to speed up the kinetics of PNP egress from A549 cells (Figure 4), as most intracellular PNP are not localized to lysosomes. Acridine orange (AO) is a nucleic acid-sensitive cationic dye often used to label DNA in cells [39,40]. However, when used in nanomolar concentrations, it labels primarily acidic intracellular organelles (e.g., lysosomes) without labeling other intracellular components (e.g., nuclei) (Figure 9a). On the basis of this characteristic, we used 7 nM AO for labeling acidic cellular organelles (mostly lysosomes), followed by detection of increased LMP index in NP-exposed A549 cells (Figure 9b). Apical NP (PNP or PM0.2) exposure caused the release of AO from lysosomes and accumulation in cytosol and nucleus. Interestingly, vesicular staining of AO did not fully disappear following NP exposure of A549 cells, indicating perhaps that the increase in LMP index was not permanent. The magnitude of NP exposure-induced increase in LMP index was comparable to that achieved by the positive control ciprofloxacin (CPX). PNP exposure in HeLa cells has been reported to result in lysosomal dysfunction via the activation of autophagy [41].

### 3.5. Absence of Mitochondrial Dysfunction in NP-Exposed A549 Cells

Mitochondria are often reported as damaged or dysfunctional in the presence of cellular stress (e.g., mild to severe loss in mitochondrial function) following exposure of cells to various NP [42]. One of the most sensitive approaches to test mitochondrial integrity is to monitor changes in fluorescence of the mitochondrial membrane potential sensitive dye TMRM in live cells. We found no appreciable changes in TMRM fluorescence intensity upon apical exposure to NP, although the positive control (i.e., FCCP) lowered TMRM fluorescence intensity by >90%. This finding might suggest that the NP dose we used in this study was too low to induce major mitochondrial impairment and that lysosomes are more sensitive than mitochondria to PNP or PM0.2 exposure at the concentration of NP used to expose A549 cells in this study.

In addition to the mitochondrial membrane potential measurement, we also utilized the novel Mtphagy dye, capable of reporting mitophagy in real time, for assessment of mitochondrial (dys)function. When mitophagy takes place (i.e., damaged mitochondria are delivered to lysosomes), the fluorescence intensity of the Mtphagy dye increases due to acidic lysosomal pH. As a positive control, FCCP treatment of A549 cells led to enhanced fluorescence intensity from the Mtphagy dye with increased colocalization of the Mtphagy dye with the LI lysosomal marker dye. By contrast, NP exposure of A549 cells failed to cause any discernible increase in the Mtphagy dye fluorescence intensity and no measurable colocalization of the Mtphagy dye with the LI lysosome marker dye, indicating no or minimal impairment in mitochondrial function.

### 3.6. Summary

In summary, we have shown in this study that PNP are taken up in part by macropinocytosis into A549 cells. Egress of PNP from A549 cells is slow and not regulated by increased cytosolic [Ca^2+^]. Intracellular presence of NP (PNP or PM0.2) activates autophagy, delivering the cargo (i.e., PNP or PM0.2) to lysosomes. Lysosomes become dysfunctional due to the presence of NP in lysosomes. NP exposure of A549 cells induces cellular stress (e.g., increased LMP index) without causing mitochondrial dysfunction(s). Further understanding of the cascade of events in cellular stress caused by NP exposure might help devise approaches to mitigation of cellular/organellar damage and pathogenesis of lung diseases (e.g., pulmonary emphysema and/or fibrosis) caused by chronic intermittent low-level NP exposure. Insights into the differences in the interactions of NP with A549 cells versus those with primary AEC may be useful in devising improved nanoparticle-based therapeutic approaches to lung cancer management.

## 4. Materials and Methods

### 4.1. Materials

PNP (20 nm diameter, carboxylated and impregnated with near infrared (NIR) dye) was obtained from Thermo Fischer Scientific, Waltham, WA, USA). Ambient air pollution particles (diameter <0.20 μm (denoted as PM0.2)) were collected from air samples in downtown Los Angeles, CA, USA, per the protocol published elsewhere [43]. Transwell filters of 10.5 mm diameter (with 0.4 µm diameter pores), fetal bovine serum (FBS), and bovine serum albumin (BSA) were purchased from BD Biosciences (Franklin Lakes, NJ, USA). A 1:1 mixture of phenol red-free Dulbecco’s modified Eagle’s medium and Ham’s F-12 medium (DME/F-12), nonessential amino acid solution (NEAA), *N*-(2-hydroxyethyl)piperazine-*N*′-(2-ethanesulfonic acid) hemisodium salt (HEPES), 2-(*N*-morpholino)ethanesulfonic acid sodium salt (MES), monodansylcadaverine (MDC), cytochalasin D (CCD), adenosine triphosphate (ATP), dimethylsulfoxide (DMSO), l-glutamine, 3-methyladenine (3-MA), bafilomycin, nocodazole, carbonyl cyanide 4-(trifluoromethoxy)phenylhydrazone (FCCP), ciprofloxacin, trypsin-EDTA, Triton X-100, paraformaldehyde, chloroquine, and acridine orange (AO) were all obtained from Sigma-Aldrich (St. Louis, MO, USA). Primocin was purchased from InvivoGen (San Diego, CA, USA). 1,2-Bis(2-aminophenoxy)ethane-*N*,*N*,*N*′,*N*′-tetraacetic acid tetrakis(acetoxymethyl ester) (BAPTA-AM) and Hoechst 33342 were obtained from Invitrogen (Carlsbad, CA, USA). Dylight (488 nm)-conjugated tomato lectin was obtained from Vector Laboratories (Burlingame, CA, USA). Some tomato lectin was labeled in-house using Dylight 405 NHS Ester labeling kit (Thermo Fischer Scientific). Fluo-8 AM was purchased from AAT Bioquest (Sunnyvale, CA, USA). BacMam LC3-GFP and Rab5a-GFP constructs, Lysotracker Green, and tetramethylrhodamine methyl ester (TMRM) were bought from Thermo Fischer Scientific. Microtubule-associated protein light chain 3B (LC3B) antibody (that recognizes both LC3B-I and -II) was purchased from Cell Signaling Technology (Danvers, MA, USA). Mitophagy detection kits (including the Mtphagy dye and lysosomal indicator (LI) dye) were obtained from Dojindo Molecular Technologies (Washington, DC, USA). A549 cells were purchased from American Type Culture Collection (Manassas, VA, USA).

### 4.2. Cell Culture

A549 cells were plated onto Transwell filters at 100,000 cells/0.865 cm^2^ and cultured in a defined medium with serum (MDS), composed of 10% FBS and serum-free defined medium (MDSF; DME/F-12 medium supplemented with 1 mM NEAA, 100 U/mL Primocin, 10 mM HEPES, 1.25 mg/mL BSA, and 2 mM l-glutamine). Cells were maintained at 37 °C in a humidified atmosphere of 95% air and 5% CO_2_ and fed every other day. Experiments were performed using A549 cells on culture days 2–3.

### 4.3. Live Cell Imaging

A549 cells cultured on Transwell filters were imaged by confocal microscopy as described elsewhere [26]. Briefly, A549 cells on Transwell filters were mounted in a temperature-controlled chamber (Vestavia Scientific, Vestavia Hills, AL, USA) and bathed with MDS on both sides. In xyz series, intracellular PNP fluorescence intensity was measured stack-by-stack and integrated over the entire volume of a single A549 cell. To demarcate intracellular space at the single cell level, cell plasma membranes were labeled using Dylight (488 or 405 nm)-conjugated tomato lectin. Confocal imaging was at 8 bits, 63× magnification, and 1024 × 1024 resolution with a SP8 confocal microscope system (Leica Microsystems GmbH, Wetzlar, Germany). Gallium nitride (405 nm), argon (488 nm), and helium-neon (633 nm) lasers were utilized for excitation. Image analysis was conducted using Image-J software (NIH, Bethesda, MD, USA) and Leica LAS 3D Process and Quantify Packages.

### 4.4. Nanoparticle (NP) Exposure of A549 Cells

PM0.2 at 1 μg/mL and near-infrared (NIR) dye-impregnated carboxylated PNP (20 nm diameter with excitation/emission (ex/em) wavelengths of 660/680 nm) at 80 μg/mL were used to apically expose A549 cells for 24 h unless noted otherwise.

### 4.5. Intracellular PNP Content Assessed after 24 h of Apical PNP Exposure

Incubation of PNP-exposed cells was performed in a humidified atmosphere of 5% CO_2_/95% air. At 24 h post exposure, cells were washed with fresh culture medium and intracellular PNP content was estimated using live cell imaging as above.

### 4.6. Effect of Endocytosis Inhibitors on Intracellular PNP Content

Intracellular PNP content was estimated by live cell imaging in the presence and absence of agents known to interfere with endocytosis pathways, including MDC (inhibiting clathrin-mediated endocytosis; 200 μM), CCD (inhibiting macropinocytosis; 10 μM), and nocodazole (inhibiting microtubule polymerization; 100 nM). A549 cells were exposed both apically and basolaterally to one of these inhibitors, beginning 30 min prior to and during apical PNP exposure of A549 cells, followed by intracellular PNP content assessment. Control represents PNP-exposed A549 cells studied in the absence of endocytosis inhibitors.

### 4.7. Egress of Intracellular PNP from A549 Cells

A549 cells were apically exposed to 20 nm PNP at 80 μg/mL for 12 h, followed by washing thrice with fresh culture medium. A549 cells were then incubated on both sides with fresh MDS for up to 24 h. Intracellular PNP content of A549 cells at predesignated times was estimated to construct egress time courses.

### 4.8. Cytosolic Ca^2+^ Mobilization and PNP Exocytosis

The effect of intracellular mobilization of Ca^2+^ on PNP egress was assessed in washed A549 cells (after 12 h of apical PNP exposure as above) stimulated with 10 μM ATP added to both bathing fluids during the egress experiment for up to 24 h. Changes in cytosolic [Ca^2+^] were detected by Fluo-8 AM (1 μM, 10 min, 25 °C in the presence of 5 mM probenecid to prevent leakage of Fluo-8 from the cells) following a brief presence (for 2.5 min) of apically added 10 μM ATP.

### 4.9. Nanoparticle Exposure-Induced Activation of Autophagy in A549 Cells

A549 cells were apically exposed to NP (PNP or PM0.2) in the presence or absence of 40 μM chloroquine, which blocks autolysosome formation, that was added to both apical and basolateral bathing fluids 30 min prior to and during NP exposure. After 24 h NP exposure, A549 cells were washed with fresh culture medium thrice and fixed with 4% paraformaldehyde for 10 min at room temperature, followed by immunolabeling of autophagosomes using rabbit LC3B antibody (1:400 dilution, overnight, 4 °C) and Alexa 488-labeled goat anti-rabbit secondary antibody (1:400 dilution, 1 h, 25 °C). Antigen-antibody complexes were detected using confocal microscopy at ex/em of 488/500–550 nm.

### 4.10. Effects of Inhibitors of Autophagosome or Autolysosome Formation on Intracellular PNP Content

Intracellular PNP content was assessed in the presence of 3-MA (5 mM, an inhibitor of autophagosome formation) or bafilomycin (0.5 μM, an inhibitor of autolysosome formation). A549 cells were pre-incubated with one of these inhibitors in both apical and basolateral fluids 30 min prior to and during apical PNP exposure.

### 4.11. Assessment of Lysosomal Dysfunction

Lysosomal membrane permeability was assessed in A549 cells labeled with acridine orange (AO, 7 nM, 37 °C and 5% CO_2_) for 15 min [40]. Lysosomal dysfunction was detected by release of AO from lysosomes due to increased lysosomal membrane permeability (LMP), followed by accumulation of AO in cytosol and nucleus. In order to quantify LMP, an LMP index was estimated as AO green fluorescence intensity in cytoplasm and nucleus/total cellular AO green fluorescence intensity. Changes in AO fluorescence intensity in A549 cells were monitored using ex/em of 488/500–525 nm. A549 cells treated with ciprofloxacin (150 μM for 24 h) in both apical and basolateral bathing fluids was used as positive control.

### 4.12. Assessment of Mitochondrial Function

A549 cells were exposed to the mitochondrial membrane potential sensitive fluorescent dye TMRM (1 nM, ex/em: 561/570–630 nm) for 30 min in culture medium at 37 °C and 5% CO_2_ prior to live cell imaging. For the detection of mitophagy, A549 cells were apically exposed to Mtphagy dye (100 nM, ex/em: 561/650–800 nm) for 30 min in serum-free culture medium at 37 °C and 5% CO_2_. After labeling A549 cells with Mtphagy dye, cells were further apically exposed to NP (PNP or PM0.2), 1 μM FCCP (as a positive control) or DMSO (0.1% as a negative control) for 24 h in MDS. Thirty minutes prior to detection of mitophagy, bathing fluids of A549 cells were changed to MDSF containing LI lysosome marker dye (1 μM, ex/em: 488/490–550 nm). Live cell imaging was performed 30 min later for evidence of mitophagy (i.e., colocalization of the lysosomal marker dye with Mtphagy dye in lysosomes).

### 4.13. Data Analysis

Data are presented as mean ± standard deviation (*n* = total number of observations). Student’s two-tailed *t*-tests were used for comparisons of two group means. One-way analysis of variance followed by post-hoc tests based on Tukey procedures was performed using Prism (version 6.07, GraphPad Software, La Jolla, CA, USA) to determine differences among means of ≥3 groups. *p* < 0.05 was considered statistically significant.

## Abbreviations

3-MA	3-methyladenine
AEC	Alveolar epithelial cell
AM	Acetoxymethyl ester
ATP	Adenosine triphosphate
CCD	Cytochalasin D
EDTA	Ethylenediaminetetraacetic acid
LMP	Lysosomal membrane permeability
MDC	Monodansylcadaverine
NP	Nanoparticle
LC3	Microtubule-associated proteins 1A/1B light chain 3B
PM0.2	Ambient air pollution particles with a diameter <0.2 μm
PNP	Polystyrene nanoparticle
RAECM	Rat alveolar epithelial cell monolayer
